# T cell immunoengineering with advanced biomaterials

**DOI:** 10.1039/c6ib00233a

**Published:** 2017-02-20

**Authors:** Derfogail Delcassian, Susanne Sattler, Iain E. Dunlop

**Affiliations:** a School of Pharmacy , University of Nottingham , NG7 2RD , UK . Email: derfogail.delcassian@nottingham.ac.uk; b Koch Institute for Integrative Cancer Research , MIT , Massachusetts , 02139 , USA; c Imperial College London National Heart and Lung Institute , Du Cane Road , W12 0NN , London , UK; d Department of Materials , Imperial College London , SW7 2AZ , UK . Email: i.dunlop@imperial.ac.uk

## Abstract

Recent advances in biomaterials design offer the potential to actively control immune cell activation and behaviour. Many human diseases, such as infections, cancer, and autoimmune disorders, are partly mediated by inappropriate or insufficient activation of the immune system. T cells play a central role in the host immune response to these diseases, and so constitute a promising cell type for manipulation. *In vivo*, T cells are stimulated by antigen presenting cells (APC), therefore to design immunoengineering biomaterials that control T cell behaviour, artificial interfaces that mimic the natural APC-T cell interaction are required. This review draws together research in the design and fabrication of such biomaterial interfaces, and highlights efforts to elucidate key parameters in T cell activation, such as substrate mechanical properties and spatial organization of receptors, illustrating how they can be manipulated by bioengineering approaches to alter T cell function.

## Abstract 2

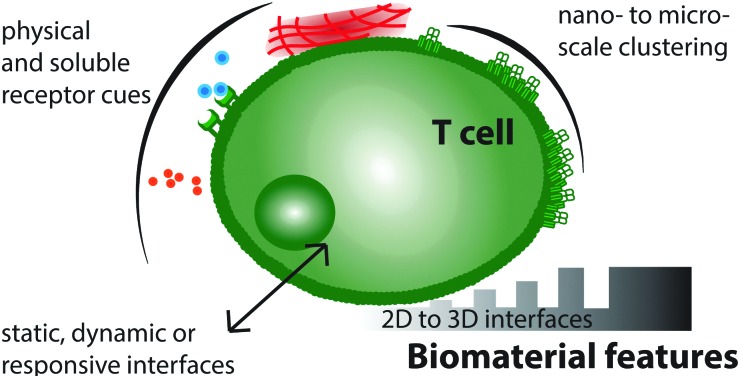
Biomaterial interfaces can present soluble and physical cues on the nano- and micro-scale to control T cell behaviour.

##

Insight, innovation, integration

Controlled immune cell activation is emerging as a powerful strategy in a range of clinical therapies. This review describes current progress in designing biomimetic structures that direct T cell behaviour. This new field integrates fundamental research in immune cell biophysics, including receptor nanopatterning and mechanotransduction, together with applied biomaterial design. We comprehensively summarize the current state of the field and advocate a future direction for innovative immunomodulatory biomaterials through the integration of spatial, temporal and mechanical cellular cues in 2- and 3-dimensional therapeutics.

## Introduction

Traditionally, biomaterials have been deliberately designed to minimize interactions with the host immune system when implanted. The desire for “immune inert” materials that avoid *in vivo* immune interactions, such as inflammation,^1^ fibrosis and encapsulation^2^,^3^ and ultimately rejection^4^–^6^ has been a major focus of tissue engineering over the last few decades. In contrast, there is a recent growth of interest in materials that achieve the opposite effect; directly influencing immune cell behaviour to control immune cell growth and differentiation, regulate inflammation or induce tolerance; effectively developing engineered materials that can direct immune cell behaviour.^7^–^9^

The development of such immunoengineering biomaterials is still a nascent field; however its rise has coincided with the rapid development of biotechnology techniques that direct the immune response. Prominent examples of this technology are genetically engineered Chimeric Antigen Receptor (CAR) T cells which efficiently target leukemic cancers,^10^–^12^ and nanoparticle drug delivery strategies for targeted immune cell vaccination.^13^–^16^ These fields promise the design of advanced T cell therapeutics that can deliver appropriate stimuli to T cells to tackle a range of disease targets. Several key parameters controlling T cell activation and behaviour have been identified, and the design of biomaterials capable of manipulating these features presents a new approach to directing T cell behaviour.

**Fig. 1:**
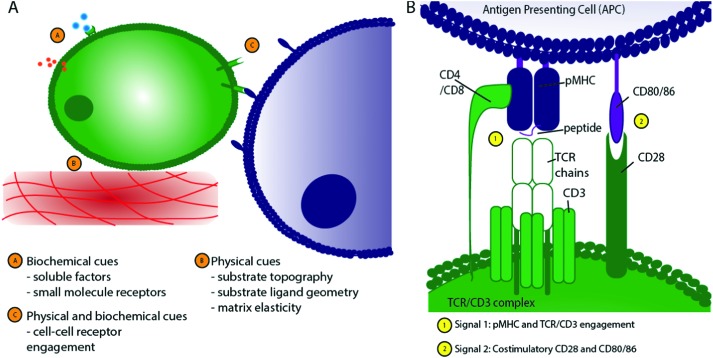
Schematic of T cell interactions. (A) T cells experience a range of stimuli *in vivo* that can be broadly classified into biochemical and physical cues. (B) The TCR/CD3 complex is central to T cell activation, requiring engagement of peptide-bearing MHC (pMHC) with the TCR/CD3 complex, CD4 on the T cell surface with pMHC on the APC cell surface, and stimulation through CD28 binding to CD80/CD86.

*In vivo*, T cells are exposed to distinct stimuli that influence their behaviour, shown schematically in Fig. 1A. These stimuli, or “cellular cues,” can be loosely classified as physical (including the topographical, geometrical and mechanical nature of the cell–substrate interface) or biochemical (including engagement of cell surface receptors and response to soluble factors). To avoid aberrant immune responses under physiological conditions, *in vivo* T cell activation is a tightly controlled process, depending on Antigen Presenting Cells (APC) providing antigen-specific signals (signal 1) and co-stimulation (signal 2), alongside the stimulation provided by the surrounding cytokine milieu (signal 3).^17^

**Fig. 2:**
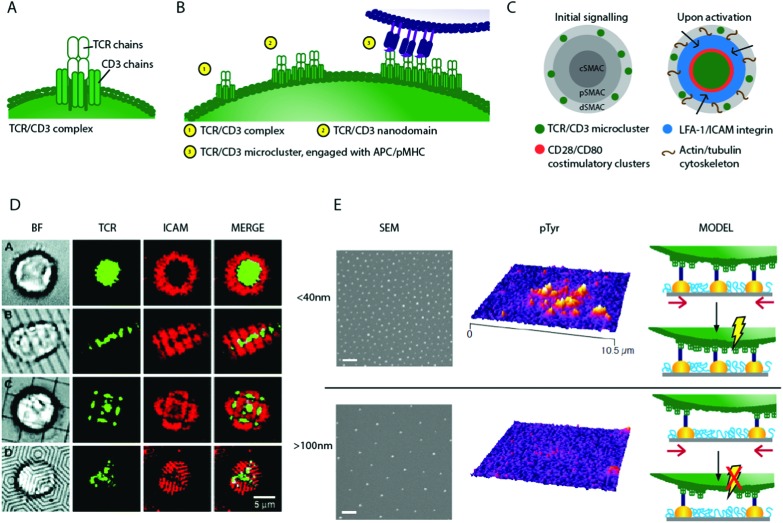
Schematic of T cell stimulation and 2D biomaterials interfaces controlling TCR movement. (A) Schematic of a single TCR/CD3 complex (B) a schematic of hypothetical micro-cluster formation; individual TCR/CD3 complexes associate to form nano-domains, which pre-exist on the T cell surface. On engagement with APC, TCR/CD3 micro-clusters form (C) a schematic of activation, where TCR/CD3 micro-clusters move from the periphery to a central Super Molecular Activation Cluster (c-SMAC), and become surrounded by rings of costimulatory and adhesion molecules. (D) Using chromium barriers on fluid lipid bilayers restricts micro-cluster formation and so synapse structure^46^ (E) nanopatterns fabricated using block copolymer micellar lithography present anchoring surfaces for TCR/CD3 ligands with precise inter-ligand spacing (scale bar 100 nm) such surfaces show dramatic differences in their ability to activate T cells. This may be due to bound nanoclusters being spaced too far apart on surfaces with large inter-ligand density, with nanoclusters unable to concatenate if pinned to static nanoparticles, preventing TCR/CD3 micro-cluster formation and so signalling, shown schematically (altered from ref. 39). Copyright: (D) adapted from ref. 46 Mossman *et al.*, *Science*, 2005, **310**(5751), 1191–1193. Reprinted with permission from AAAS; (E) adapted with permission from ref. 39 Delcassian *et al. Nano Lett.*, 2013, **13**(11), 5608–5614, Copyright 2013 American Chemical Society.

Of particular importance to appropriate T cell activation is the formation of a closely-adhered immunological synapse between the T cell and APC (Fig. 2B and C) where a large number of ligand–receptor pairs combine to form a complex structure^18^,^19^ that determines the T cell's activation and downstream differentiation. Peptide-bearing Major Histocompatibility Complex (pMHC) presented by APCs engages with the T cell receptor (TCR) on T cells to create an initial activation signal. Secondly, a co-stimulatory ligand–receptor pair, such as surface receptors CD28/CD80, is engaged to support full activation, as shown in Fig. 1B.

Additional signals, such as those provided by the cytokine milieu, provide the third signal to allow effector T cells to expand and exhibit their full function.^5^,^20^ This tightly regulated stimulation and co-stimulation is crucial to maintaining healthy T cell function. Without efficient co-stimulation, antigen presentation can lead to T cell anergy, while inappropriate response to one or more of these signals may cause hyperinflammatory conditions, such as allergy or autoimmunity.^21^ Signal 1, where the T Cell Receptor (TCR)/CD3 complex recognises the pMHC on APCs, is central in initiating T cell activation. Alternatively, in the absence of pMHC, anti-CD3 can be used to engage the TCR/CD3 complex and can provide a non-antigen specific stimulus as signal 1. Most research into modulation of T cell function by biomaterials has focused on TCR- or CD3-mediated activation.^22^–^24^ The precise physical nature of pMHC presentation to the TCR is critical for T cell activation; factors including agonist valency,^25^–^29^ the mechanical properties of the activating surface^30^,^31^ and the spatial organisation of stimulatory ligands^32^–^37^ have been identified as key features modulating T cell function in response to stimulation.

Recently, a number of research groups have attempted to explore the ability of these variables to control cellular behaviour, using advanced biomaterials that replicate specific properties of the APC surface. Studies have explored a wide range of parameters, including spatial structuring by both photolithography and self-assembly methods, and the use of bio-functionalized hydrogels and rubbers to vary mechanical properties. Here, we consider materials that are designed to directly explore these parameters, and their effects on T cells. We will examine both 2D and 3D biomaterials, and consider them in order of increasing complexity. Specifically, we will focus first on materials that present a static surface to the cell, secondly on those that are responsive (interfaces the cell can manipulate or rearrange) and finally we discuss the move towards substrates that can dynamically manipulate cellular behaviour in response to external stimuli, summarized in Table 1. This focused review draws together these experiments to provide a new perspective on the design of immunomodulatory materials, and summarizes work completed so far in the move towards “immune directing” biomaterials for the control of T cell function.

**Table 1:** Complexity in T cell biomaterials design and classifications. An overview of the parameters currently explored in T cell advanced biomaterials design, and parameter combinations that have not yet been studied. Interfaces are classified as static (fixed stimulatory substrates), responsive (cells can remodel substrates) or dynamic (substrates can be altered based on external stimuli to instruct cells). Cellular cues can be either chemical or physical, occurring in 2D or 3D, and on the micro- or nanoscale. Example references are included to illustrate some of the research performed in these areas

Class	Description		Micro-scale	Nano-scale
Static	Chemical	2D	32 and 38	39–42
		3D	43	44
	Physical	2D	31 and 45	
		3D		

Responsive	Chemical	2D	36	46 and 47
		3D	48 and 49	
	Physical	2D		
		3D		

Dynamic	Chemical	2D		
		3D	50	51 and 52
	Physical	2D		
		3D		

## Biomaterials for T cell activation

### 2-Dimensional interfaces

The region of contact between an immune cell and an antigen presenting cell (APC) is termed the immunological synapse. Usually this interface includes known activatory, inhibitory or adhesion molecules arranged in an organised manner.^19^,^53^–^55^ *In vitro*, this interaction can be replicated by creating advanced biomaterials that replicate specific properties of the APC surface, functionalized with ligands that bind specifically to known receptors on the immune cell surface. These materials can be used to form an artificial model of the immune synapse that can engage with cells. Here, we review artificial interfaces that explore the spatial and physical presentation of T cell ligands in 2D.

#### Spatially-controlled presentation of biomolecular cues

The spatial organisation of cell surface proteins at immune synapse interfaces, and the dynamic organisation of these proteins, is a central part of immune cell signalling.^18^,^54^ T cells have many proteins and lipids organised into nanoscale molecular clusters (nanodomains) on their cell surface, these contain a collection of distinct receptors and signalling molecules including the TCR/CD3 complex, and signalling molecules Lat, Zap-70 and SLP-76.^56^–^58^ Upon activation, microclusters, each containing several nanodomains, move across the cell surface to form Super Molecular Activatory Clusters (SMACs) containing several distinct protein domains (Fig. 2C).

The movement of protein and lipid molecules across the cell surface into central c-, peripheral p- and distal d-rings surrounding a central feature point is a key step in T cell activation mediated by the intracellular cytoskeleton.^59^–^65^ Early artificial immune synapses used fluid lipid bilayers to explore the formation and movement of these dynamic protein domains.^33^,^66^ Fluid lipid bilayer substrates are coated with phospholipid molecules which mimic the natural bilayer found in the cell membrane, and can be loaded with proteins that can engage with cell surface receptors. On activation, the cell may rearrange proteins expressed on the cell surface into activatory clusters, causing the proteins embedded in the fluid bilayer, and those engaged with receptors on the cell surface, to be redistributed. This allows the cell to re-arrange the proteins embedded in the fluid bilayer interface. To explore activation through the TCR, pMHC molecules specific to the cognate TCR can be introduced into the lipid bilayer. Alternatively, anti-CD3, which does not require a specific match to bind to the TCR/CD3 complex, can be used. By using these TCR agonists in conjunction with advanced microscopy techniques, the formation and movement of individual TCRs and TCR protein micro-clusters during cellular activation have been explored.

These studies showed that during T cell activation, TCR/CD3 micro-clusters form in the p-SMAC and are transported to the c-SMAC,^60^,^67^ as illustrated in Fig. 2C. The movement of TCR clusters across the cell surface to the c-SMAC on activation is now known to propagate and aid sustained signalling in T cells, and is thought to be mediated by F-actin^61^,^68^ and myosin II.^69^ By adding nanoscale chromium barriers to the fluid bilayer, more advanced substrates prevented the movement of TCRs in certain directions (Fig. 2D). Through directing and constraining the TCR micro-cluster mobility, signalling in the cell can be controlled; signalling is maximised if TCR micro-clusters are constrained to the p-SMAC using these devices.^46^,^70^ In an alternative approach, synthetic polymer chains which present anti-CD3 and anti-CD28 at specific sites on a semi-flexible poly(isocyano peptide) polymer chain have been used to activate T cells. The polymers present multiple copies of the antibodies on a single chain, and so enable multivalent receptor interactions. These are thought to lead to surface receptor rearrangements and activation of the T cell.^71^

Intriguingly, T cells display distinct patterns of micro-cluster proteins both before and during activation.^72^,^73^ These patterns typically demonstrate a TCR/CD3 enriched protein domain in the c-SMAC surrounded by a ring dominated by adhesion receptor LFA-1. The TCR/CD3 domains may display a bulls-eye or multi-focular pattern within the LFA-1 domain, depending on cell phenotype, maturity and activation status.^32^,^36^,^74^–^76^

To explore the importance of these micro-features, and the ways in which changes in protein distribution can control cellular activation, planar substrates with a range of protein micropatterns have been fabricated by microcontact printing. In one example, anti-CD3 was micropatterned in a focal or annular patch, and the activation response of T cells on these substrates measured by cytokine secretion. Much higher secretion of the cytokine IFN-γ occurred on patterns with focal anti-CD3 protein organisation compared to annular rings, even when the amount of antigen presented was the same. The inclusion of co-stimulatory agents such as anti-CD28 augmented activation, however positioning anti-CD3 and anti-CD28 in separate distinct focal points (rather than a singular focal point patterned with both stimuli) resulted in much higher IL-2 production.^47^ Separately, recent work has shown that regulatory T cells (Treg) display a reduced sensitivity to micropatterns in comparison to effector T cells,^76^ whilst functionally distinct subsets of CD4+ effector T cells (Th1 and Th2) form distinct patterns in their immunological synapse with APCs.^74^,^75^

Clearly, T cell activation is partly driven by spatial TCR micro-cluster distribution and movement; thus a logical next step explored whether controlling the dynamics of formation of these TCR micro-clusters could control T cell behaviour. Many studies indicate that smaller TCR nano-domains exist prior to ligand engagement, and that these nano-domains may play a significant role in the process of micro-cluster formation. Super-resolution microscopy has been used to explore the size of these pre-clustered TCR nano-domains on the surface of T cells; individual TCR complexes (10–20 nm in size) have been recorded^24^ and TCR nano-domains of 35–70 nm in size have been reported on non-activated T cells.^56^,^60^,^77^–^80^ On activation, these nano-domains are hypothesized to associate, transitioning from individual nanoscale units to larger structures, as illustrated in Fig. 2B.^81^ Indeed, such pre-clustering of TCR complexes may facilitate T cell activation by easing micro-cluster formation, and similar pre-clustering is also seen in other immune cell types.^79^,^80^,^82^–^86^

To explore the relationship between individual TCR receptors, potential pre-clustered nano-domains and micro-clusters, several research groups developed nano-interfaces fabricated using the self-assembly-based block copolymer micellar lithography technique. This allowed the precise positioning of gold nanoparticles (Fig. 2E) with controllable inter-particle distance that were subsequently functionalised with TCR agonists such as anti-CD3 or pMHC. Using this fabrication technique, we and others showed that early stage activation in human and mouse CD4+ T cells was a function of inter-particle spacing.^39^–^42^ As inter-particle spacing was increased from 25 to 100 nm, activation decreased, and above an inter-ligand threshold of approximately 100–150 nm, T cells were unable to activate despite the presence of stimulatory receptors. These observations are in line with the biological observation that triggering full T cell activation requires activation signals to exceed a certain threshold, in which TCR ligand density plays a key role.^25^,^26^,^87^,^88^ There are several potential explanations for this observation; primarily we consider a minimum density of receptor engagement, receptor cluster concatenation and/or minimum force requirements to be likely candidate models.

Fig. 2E illustrates one hypothesis; that pre-clustered TCR nano-domains are required to concatenate before micro-cluster formation, transport and sustained signalling can occur. If receptor nano-domains are bound too far apart, they are unable to concatenate and induce signalling. This is supported by the observed spacing activation threshold of ∼70 nm, corresponding well to the nano-domain length scale of 35–70 nm as measured by Lillemeier *et al.*^81^ Further supporting the role of clustering in cellular activation, interfaces fabricated using a single walled carbon nanotube composite have been used to activate T cells. Nanotubes coated with anti-CD3 protein and embedded in a polymer matrix enhanced T cell activation compared to cells stimulated using soluble agonist at the same concentration.^89^–^91^ The induction of efficient T cell proliferation for immunological assays has long relied on coating anti-CD3 and anti-CD28 onto standard tissue culture plates or onto beads to induce TCR crosslinking, as soluble antibodies do not induce efficient activation, suggesting that pre-clustering antibodies enhances stimulation.

A separate hypothesis considers engagement of a minimum number of receptors within a particular sub-cellular location, which may be required to allow signalling and stimulation to overcome the intrinsic activation threshold in immune cells necessary to protect from aberrant activation.^92^,^93^ Although receptor–receptor proximity, and even the proximity of several different receptor types to one another, clearly plays an important role in controlling cellular activation, such spatial effects are intrinsically coupled to receptor and cytoskeletal mechanics. In positioning receptor ligands precisely, nano- and micro-patterned interfaces also alter the position and number of traction anchoring points for the cell. This means that the observed effects of nanoscale ligand patterning could arise directly from the mechanical activation of the TCR, hinting that T cell stimulation requires a certain force to be applied through each TCR to allow conformational changes, and activation, downstream of TCR engagement.^20^,^31^,^37^,^63^,^94^,^95^ Interestingly, an intact actin cytoskeleton appears to be crucial for T cell–APC interactions; without actin, several receptors are unable to form the described micro-clusters, and protein rearrangement crucial for signalling cannot occur.^59^,^62^ Both F-actin and myosin IIA are thought to play a key role in the movement of cell surface proteins during TCR activation, and the dynamic polarisation/depolarisation of intracellular actin is key to the directional formation of the SMAC.^59^,^61^,^68^,^69^ Despite these observations, the nature of the dynamic relationship between applied mechanical force, cytoskeletal rearrangement, TCR stimulation and the level of downstream cell activation is not yet fully elucidated.^96^,^97^ In order to fully understand such effects, more complex interfaces that orthogonally vary ligand patterning and substrate mechanical properties are needed. Typically, silicon or glass materials have been used as underlying substrates for the fabrication of advanced micro- and nano-patterned interfaces, however neither material facilitates modulation of substrate mechanical properties in a biologically relevant range. The next section discusses biomechanical aspects of T cell biomaterial interface design, including the mechanical properties of biomaterial substrates.

#### Biomechanics of T cell interface design

Several cell types have been shown to respond to alterations in the mechanical properties of their environments^30^,^98^–^102^ however it is only recently that these investigations have been extended to immune cells. A number of immune receptors have been identified as mechanosensitive, where engagement of receptors with their cognate ligands is sensitive to the force applied through each bond, with the leukocyte catch bond in LFA-1–integrin binding being a well-established example.^37^,^103^–^106^ Intriguingly, TCR–ligand affinity has been shown to play a major role in determining cellular activation levels. During thymic development, T cells with high affinity interactions between their TCR and self-antigen bearing pMHC are deleted to generate a T cell repertoire with low affinity for self-antigens (>500 μM) and higher affinities for pMHC clusters with foreign peptides (1–10 μM), helping to prevent autoimmunity.^22^ During an immune response, high and low affinity interactions between the TCR and pMHC lead to different functional phenotypes,^107^,^108^ highlighting the ability of the TCR to respond to affinity, and perhaps force, through the TCR.

To explore the role of rigidity on cellular activation, biomaterial surfaces with varied mechanical properties have been fabricated. Using planar poly(dimethyl siloxane) (PDMS) substrates functionalised with stimulatory anti-CD3 antibodies, maximal proliferation in CD4+ and CD8+ T cells has been observed in cells cultured on surfaces with a rigidity of <100 kPa compared to 2 MPa.^30^ However, other studies on poly(acrylamide) (PA) surfaces describe maximal T cell responses on substrates with rigidity of 200 kPa^31^ compared to softer substrates at 10 kPa. Separately, B cells showed preferential activation on PA gels at around 20 kPa (compared to softer 2 kPa substrates)^98^ and hematopoietic stem cells demonstrated increased cell spreading on much stiffer PA substrates^100^ of around 200 kPa compared to less rigid matrices. The differences in these results pose interesting questions on how cells sense rigidity, and whether various material chemistries may play a role. It has been suggested that cells may “feel” the rigidity of the chemical bond connecting the ligand of interest to the substrate^109^,^110^ rather than the rigidity of the substrate itself.

Force transduction by the TCR/CD3 complex has been investigated using flexible micropillars coated with anti-CD3 to measure forces applied by the cell to the substrate.^111^,^112^ These experiments showed that T cells apply forces *via* the TCR/CD3 complex to such bio-functionalized substrates, and implicitly therefore to APC. In contrast, CD28 was shown to affect force transduction only indirectly by influencing intracellular signalling, with no evidence of significant traction forces being applied through the CD28 receptor itself. TCR-mediated traction forces were of the order of 100 pN per 1 μm-diameter pillar, much smaller than the forces observed in integrin-mediated cell adhesion on similar substrates.

A plausible hypothesis to explain the mechanosensitivity of the T cell is that mechanically-induced conformational changes in TCR/CD3 are needed for activation. Such changes could be induced by cytoskeletal pulling forces.^84^ It is well known that cytoskeletal function is important to T cell function; surface bound triggering requires an intact cytoskeleton,^28^ and changes in the actin cytoskeleton compromise signalling in T cells as discussed earlier, supporting this hypothesis.^37^,^59^,^62^–^64^,^113^,^114^

In several receptor types, such as selectins, there is a transition between catch and slip bond systems at specific force values, implying that the cell can sense force over a dynamic range and that force can regulate conformational changes in these systems.^105^,^106^ *In vivo*, these mechanisms are crucial for immune cell extravasation from blood vessels to allow migration into inflamed tissues. Immune cell extravasation is largely dependent on L- and E/P-selectin adhesion receptors, which allow leukocytes to first tether and roll along the activated endothelium and then firmly adhere before squeezing into the tissue through gaps between endothelial cells. Stabilisation of leukocyte adhesion to the blood vessel wall requires a minimum level of fluid shear stress in order to avoid inappropriate aggregation or adhesion outside the vasculature. This has been called catch–slip transition and explains how increasing levels of shear stress and thus force applied to a specific receptor–ligand pair, can stabilize leukocyte adhesion under flow.^115^–^117^

A similar mechanism could occur within the TCR, for example exposing cryptic binding sites downstream of the TCR/CD3 machinery that are force sensitive, using a similar mechanism to cellular force-sensing in the formation of focal adhesions.^118^,^119^ Additionally, the mechanical force applied through the TCR could correlate with viral or infectious load, so that the number of anchoring points and/or force loading provides the T cell with additional information about the status of an APC. Intriguingly, studies have demonstrated a link between the spatial distribution of proteins on T cell surfaces, the intrinsic mechanical properties of T cell, and the activation level of the cells.^120^

Importantly, a ‘receptor deformation model’ has been proposed, which provides an explanation for TCR triggering under physiological conditions, where the amount of one specific peptide available is likely too small to allow activation based on TCR crosslinking.^94^,^95^ The model suggests that the pulling force associated with T cell motility induces a TCR conformation that favours induction of downstream signalling. To fully explore these mechanisms, advanced materials that vary rigidity simultaneously with other variables (such as ligand chemical linkage and spatial nanopatterning) are required.

### 3-Dimensional interfaces

The natural T cell–APC interaction occurs in three dimensions (3D) between two cells which are themselves 3D objects. In addition to their overall shape, natural APCs and immune cell surface membranes exhibit a range of micro topographical features including invaginations, protrusions, and extending nanotubes.^121^,^122^ Given the inherently 3D nature of immune cells, and the wealth of 3D features present on their surfaces, more complex biomaterial interfaces are starting to be developed to explore the cellular response to altering these features. Here, we describe interfaces that replicate the 3D aspects of cell surface features, and move towards more complex systems.

#### Topography

Dendritic cells, the prototype APC, are known to possess distinct lamellipodea, and are often characterized in cartoons as “spiky cells”. Despite this, there has been limited study into the influence of these micro- and nano-scale features on T cell activation. The dendritic cell protrusions may be a strategy for increased cell membrane surface area to cell volume ratio, allowing for an increase in pMHC available on the APC surface, or alternatively, could provide a varied topography required for activation.

Using advanced microfabrication techniques, micro-topographical features have been patterned on planar substrates to explore their effects on T cell stimulation. Large micro-pits have been used to trap individual T cells^122^ and investigate the morphology of the APC interface, however studies into the effect of topography itself are extremely limited. Although the effect of surface topography on T cell behaviour has not been well studied, evidence from other immune cells suggests this may be an important parameter to consider in stimulatory biomaterial interface design. Neutrophil activation is enhanced on budding, as opposed to smooth, microparticles.^123^ Similarly, surface topography has been shown to induce phenotypic shifts towards distinct subtypes in macrophages; titanium surfaces with smooth or micro pitted features were able to induce macrophage differentiation towards an “inflammatory” or “regenerative” phenotype, respectively.^124^

#### 3D architecture

To more accurately replicate the 3D interaction between T cells and APCs, a range of spherical micro- and nano-particle materials have been designed to boost T cell activation. Many of these materials are primarily designed as vaccine therapies, which aim to induce enhanced immune responses through the delivery of antigen to dendritic cells or lymphoid organs. This antigen is then processed and presented to T cells as part of the natural pMHC complex. Separately, the closest mimic of the pMHC complex on the dendritic cell is another cell engineered to express selected agonist ligands. These biologically engineered materials possess natural fluid lipid bilayers over the surface of the cell, intact actin cytoskeletons that may be used to aid traction of force and so activation, and a range of natural adhesive ligands such as integrins. The rational design and targeted delivery of 3D spherical micro-, nano- and cellular engineering material vaccine therapies has been reviewed extensively elsewhere.^7^,^8^,^13^,^20^,^66^,^125^,^126^ In this section, we instead focus on the key principles governing the 3D design of artificial biomaterials that directly stimulate the T cell itself.

A range of materials have been used to fabricate fully synthetic spherical constructs including polystyrene,^127^ PLGA,^43^ polymer emulsions,^128^ and synthetic liposomes.^48^,^49^ These materials are usually functionalized with proteins, either chemically linked or adsorbed onto the material surface. Importantly, both the size and shape of the material play an important role in cellular activation.^13^,^126^,^129^ Non-spherical, micro-scale ellipsoid shapes have been shown to enhance T cell activation when compared to their spherical^129^ counterparts, possibly due to changes in presented surface area or T cell membrane disruption. A range of studies have explored the role of particle size in both vaccine delivery strategies and, independently, phagocytosis of nanoparticles in several immune cell types.^13^,^130^ For T cells, it has previously been shown that nanoscale particles are less effective than their microscale counterparts in stimulating T cells towards activation.^131^,^132^ Particles of at least 4–5 μm diameter are thought to be required to provide the minimum contact area^133^ needed for full activation. The reasons for this are unclear, but are likely to include the need for microscale contact areas to enable receptor segregation (SMAC formation), or the inability of smaller interfaces to establish long-range mechanical coupling across distinct parts of the T cell as discussed in other sections of this review.

Spherical interfaces with diameters above this 4–5 μm threshold are therefore strong potential candidates for *in vivo* therapeutic applications, and indeed polystyrene beads bio-functionalized with anti-CD3 and anti-CD28 have been used to stimulate T cells in clinical trials.^12^,^134^ Moving to more flexible self-assembled materials, PEGylated liposomes have recently proven successful in targeting up to 95% of circulating adoptively transferred T cells^48^,^49^ offering great promise for future *in vivo* therapies using multicomponent systems that are able to engage selectively with target T cell sub-populations.

Interestingly, recent work in this area has outlined more advanced materials for stimulation in 3D; Janus particles^135^ presenting focular CD3 protein micropatterns have been used to stimulate T cells representing a move towards 3D materials with controlled surface patterning. The design of 3D interfaces that move from simple, single parameter interfaces towards more complex architectures that consider several parameters at a time will be the next step in T cell biomaterial design.

### Dynamic interfaces

Natural T cell–APC interactions are dynamic in nature, with distinct events occurring over a timescale that starts with cellular stimulation in the first few seconds, followed by a complex cascade of activation events occurring over several hours.^136^–^138^ Fully interactive innate and adaptive immune responses often occur over several months, and in some cases, protective immune response can last a lifetime. The sequence, duration and nature of each step in the stimulation pathway can drastically affect cellular outcome. In moving to more advanced biomaterial interfaces, it will be important to develop materials that can dynamically control cellular behaviour. These materials should controllably alter their properties in response to externally applied stimuli, and therefore provide a range of cues to the cell that are conditioned to specific environments, effectively modulating T cell behaviour.

**Fig. 3:**
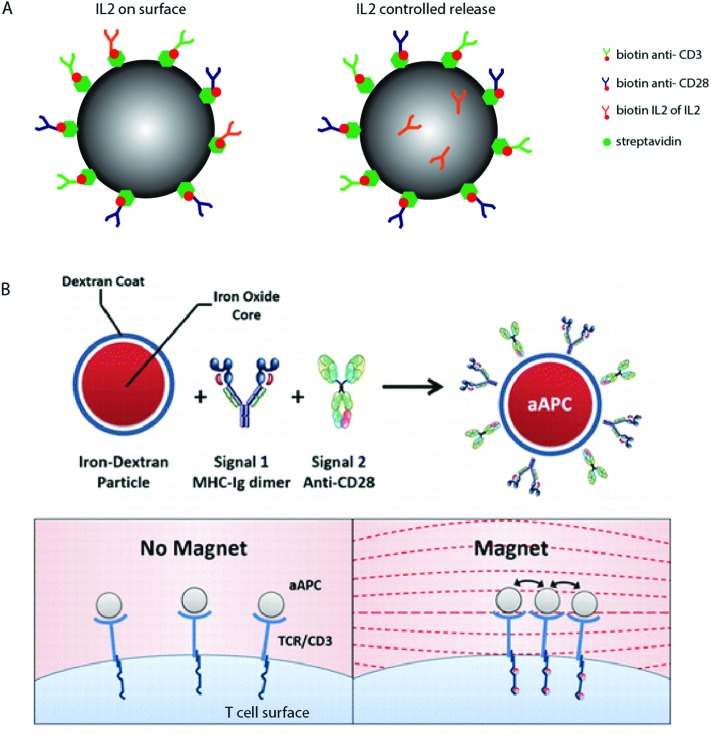
Dynamic T cell interfaces with APC-mimicking biomaterials. (A) Biodegradable PLGA particles have been used to deliver surface bound and soluble T cell signals, paracrine IL2 delivery seems to result in increased activation.^50^ (B) A schematic of paramagnetic nanoparticles functionalized with anti-CD3 and anti-CD28, which represent the move towards dynamic biomaterials that are able to induce T cell activation in response to specific stimuli. Copyright: (B) adapted with permission from ref. 51 Perica *et al. ACS Nano*, 2014, **8**(3), 2252–2260, Copyright 2014 American Chemical Society.

An example includes the temporal delivery of stimulatory signals to T cells, explored using biodegradable polymers. Fig. 3A shows a schematic where polymer particles have been functionalized with anti-CD3 and anti-CD28, and modified to contain the cytokine IL2 that is either chemically linked to the particle surface or encapsulated within the polymer matrix and released as the polymer degrades.^50^

These materials have shown that paracrine release of IL2 results in enhanced T cell activation compared to materials that present IL2 directly on the surface. *In silico* experiments which model the release of protein and cytokines from biodegradable materials^50^,^132^ suggest that the kinetics of release may drastically affect cellular behaviour.

In other fields, the manipulation of material physical properties using external stimuli, such as temperature, pH, or UV light, has been widely explored as a tool to regulate kinetic drug dosing, ligand presentation and subsequent cell behaviour^139^–^141^ however these studies are limited in respect to T cell stimulatory biomaterial design. One notable example involves the manipulation of TCR nanoclusters using functionalized para-magnetic particles.^51^ These particles bind to TCR receptors on the cell surface, and naturally aggregate in an applied magnetic field. The aggregation of these particles induces TCR clustering, which leads to magnetically-induced cell activation, shown schematically in Fig. 3B. We anticipate that using a similar principle, TCR aggregation (and so activation) could be induced using a range of stimuli response biomaterials in future dynamic artificial immune synapse design.

## Conclusions

The biomaterials described here exemplify a paradigm shift in biomaterial design away from traditional “immune inert” materials towards materials that are specifically designed to control, and perhaps elicit, specific immune cell behaviour. A range of factors controlling T cell behaviour, such as spatial patterning, substrate geometry and substrate mechanical properties have been identified and individually explored using increasingly complex static, responsive and dynamic biomaterial substrates. Table 1 highlights the breadth of materials that have already been fabricated to explore these parameters, but also indicates that there is huge scope for development of more complex materials capable of supporting artificial immune synapses, with many possible parameter combinations as yet unaddressed.

The high level of interest in T cell biomaterial design arises not only from the fundamental importance of modulating T cell activation in human biology but also from the potential of these materials to contribute to current therapeutic aims. Determining the mechanisms that underpin phenotype specific induced activation pathways will have important implications in future immunotherapeutic design. The long-term aim is for advanced biomaterial interfaces to facilitate the generation or delivery of precisely-controlled T cell populations of desired phenotypes for each specific therapeutic application.

Static biomaterial interfaces, including nanopatterned and rigidity-controlled substrates, present precisely-controllable stimuli designed to induce a specific response in T cells, and have delivered fundamental insights into the thresholds for different parameters to stimulate T cells. Observations from these works highlight that the nano- and micro-patterned distribution of TCR/CD3 ligands on stimulatory surfaces play a key role in T cell activation, with signalling thresholds directly related to the precise positioning of TCR/CD3 complexes and co-stimulatory receptors.

Other studies have focused on the role of substrate mechanical properties in controlling T cell activation. These studies are limited in number, but indicate that force appears to affect activation through the TCR/CD3 complex. It is important to note that many of the variables investigated, including ligand–receptor spacing and protein patterning, may be intrinsically linked to adhesion force. To fully understand the importance of these parameters, more complex biomaterials will be needed to isolate and re-combine them, allowing an exploration of the relative contributions of each of these factors.

Responsive materials present stimuli to cells, but can be remodelled by the responding cells to allow modulation of behaviour driven by the cell. An important example is the fluid lipid bilayer system, which has provided a wealth of information about the rearrangement of ligands following activation. A variety of ligand protein distribution sizes and patterns have been observed on the cell both before and during activation. Moving forward in the design of immune cell–biomaterial interfaces, it will be important to understand the nature of these differences, and whether they may be due to the behaviour of distinct T cell phenotypes.

Finally, dynamic materials are those that respond to external stimuli to induce a specific state in the cell only under certain conditions. Research on dynamic biomaterials for the manipulation of immune cell behaviour is still in its infancy, but offers great promise, using varying kinetic and stimuli responsive biomaterials to fine tune immune responses and potentially enable localized, rather than systemic, control of immune behaviour. Coupled with cell engineering strategies, these materials could enable a more complex temporal and spatial regulation of immune cell behaviour to deliver individually engineered immune therapies.
